# Supplementary material for: Clinically relevant molecular subtypes and genomic alteration-independent differentiation in gynecologic carcinosarcoma
Source: Nat Commun. 2019 Oct 31;10:4965. doi: 10.1038/s41467-019-12985-x (PMC6823358; doi:10.1038/s41467-019-12985-x)
Supplement: Supplementary file 1 — Supplementary Information [file 41467_2019_12985_MOESM1_ESM.pdf]

Supplementary Information

Clinically Relevant Molecular Subtypes and Genomic Alteration-Independent Differentiation in Gynecologic Carcinosarcoma

Osamu Gotoh[1], Yuko Sugiyama[2], Yutaka Takazawa[3], Kazuyoshi Kato[2], Norio Tanaka[1], Kohei Omatsu[2], Nobuhiro Takeshima[2], Hidetaka Nomura[2], Kosei Hasegawa[4], Keiichi Fujiwara[4], Mana Taki[5], Noriomi Matsumura[5], Tetsuo Noda[1], Seiichi Mori[1*]

Supplementary Note 1

Study Design and Sample Characteristics

We conducted integrated genomic, transcriptomic, and epigenetic analyses of uterine and ovarian CS samples taken from clinical specimens harvested during surgery for the treatment of CS. Clinicopathological features, such as anatomical site, carcinoma and sarcoma histology, stage, grade, and other patient variables are shown in Supplementary Figure 1 and are described in the Supplementary Materials and Methods. Custom panels were designed by selecting genes based on significance in gynecological malignancy, sarcoma development, actionable targets, and DNA repair (see details in the Methods).

Beginning with 142 uterine and 27 ovarian CS primary tumors, a final total of 109 (92 uterine and 17 ovarian) samples passed the stringent quality assessments at multiple stages of pathological review, sample preparation, sequencing, and informatics analyses for targeted re-sequencing or exome sequencing, as shown by the REMARK Diagram in Supplementary Figure 2.

Supplementary Note 2

Transcriptomic and Epigenetic Subtypes in CS

The biological and clinical relevance of the molecular subtyping based on the genomic aberration profile prompted us to examine whether internal subtypes could be distinguished using other modality data. We performed consensus clustering (CC) using transcriptome and DNA methylome data for primary tumors, and compared the resultant subtypes with the clinicopathological attributes of the patients and with the results from the other subtyping method (Supplementary Figures 7 and 8). CC with highly variably expressed genes (top 14% selected by "pvclust") analyzed using RNA-seq data led to the identification of 5 transcriptomic subtypes (TSs). TS1 was characterized by epithelial marker expression and mitochondrial gene expression. TS2 was characterized by a high level of epithelial marker expression and a high level of immunoglobulin gene expression. Genes related to angiogenesis, metastasis, and epithelial-mesenchymal transition (EMT) were enriched in TS3. TS4 and TS5 featured moderate expression of epithelial and neuronal genes, respectively (Supplementary Figure 7). The proportion of genomic aberration subtypes and the rate of carcinoma-sarcoma components differed across the TSs and were linked with patient outcomes (Supplementary Figure 7). Following this, CC using CpG methylation profiles derived from DNA methylation microarrays revealed 4 epigenetic subtypes (ESs). Whereas ES1 and ES2 clusters were characterized by intermediate- and hypermethylated CpG probes, respectively, ES3 and ES4 were hypomethylated. ES1 and ES2 coincided with a mix of POLE and MSI molecular subtypes as well as with *MLH1* promoter hypermethylated samples, and tumors of the CNH subtype were almost exclusively distributed to ES3 and ES4 (Supplementary Figure 8). The ESs were significantly correlated with patient prognosis (Supplementary Figure 8). These internal subtypes found in the transcriptome and DNA methylome thus correlated with the clinicopathological features and with the subtypes derived from the other modality data.

Supplementary Note 3

Genetic Events Possibly Promote Differentiation between Carcinoma and Sarcoma

As described in the “Evolutionary Analysis of Carcinoma and Sarcoma Components with Differential Sampling” and “Genetic Similarity of Multiple Regions with Variable Composition of Carcinoma and Sarcoma Elements in a Tumor” in the Results (Supplementary Table 4 and Supplementary Data 5 and 6, and Figures 5 and 6), we found that most of the clonal genetic driver events occurred on the trunk of the phylogenetic tree (target panel and exome sequencing analyses). To identify any differential SNV/indel or aberrant copy number segment (detected in GISTIC analysis), we further examined both the target panel and exome sequenced results derived from carcinoma and sarcoma elements that were dissected separately (Supplementary Table 4 and Supplementary Data 5 and 6). In the case of exome data analysis, we examined all genes for which any variant was detected. However, we did not identify any recurrent genetic events that could account for differentiation of carcinoma and sarcoma cells (Supplementary Data 5 and 6). Similarly, there were no genetic events identified through comparisons of SNVs/indels and CNVs between carcinoma- and sarcoma-dominant regions with multi-regional exome sequenced data of three cases (EN676, GY030 and EN558; the data from EN482 were not used for this purpose since sarcoma-dominant region sequenced data was not available for the case). Thus, overall, neither our target panel nor exome sequencing analysis could detect any relevant genetic events in the differentiation of carcinoma or sarcoma.

Supplementary Note 4

EMT Score, *CTNNB1*-activating Mutation, and Methylation Status of miR-200a/200b/429 and miR-141/200c

A recent paper reported enrichment of *CTNNB1*-activating mutations in the CNL subtype of endometrial endometrioid carcinomas using TCGA data [1]. The transcriptome analysis also revealed β-catenin–activating mutations leading to EMT-related gene transactivation in endometrioid cancer samples, which is consistent with observations in other cell types, such as breast cancer cells [1]. However, in our CS cohort, the EMT scores for tumors with *CTNNB*-activating mutations were comparable with those of tumors lacking β-catenin mutations, unlike with the TCGA endometrioid carcinomas [1, 2]. We observed significant hypomethylation of miR-200a/200b/429 and miR-141/200c probes in tumors with *CTNNB1*-activating mutations, regardless of the genomic aberration subtype (Supplementary Figure 10). This finding implies possible negative feedback by activated β-catenin to methylation status of the microRNAs and there are distinct molecular mechanisms involved in the activation of the EMT transcriptional program between endometrioid carcinoma and carcinosarcoma.

Supplementary Figures

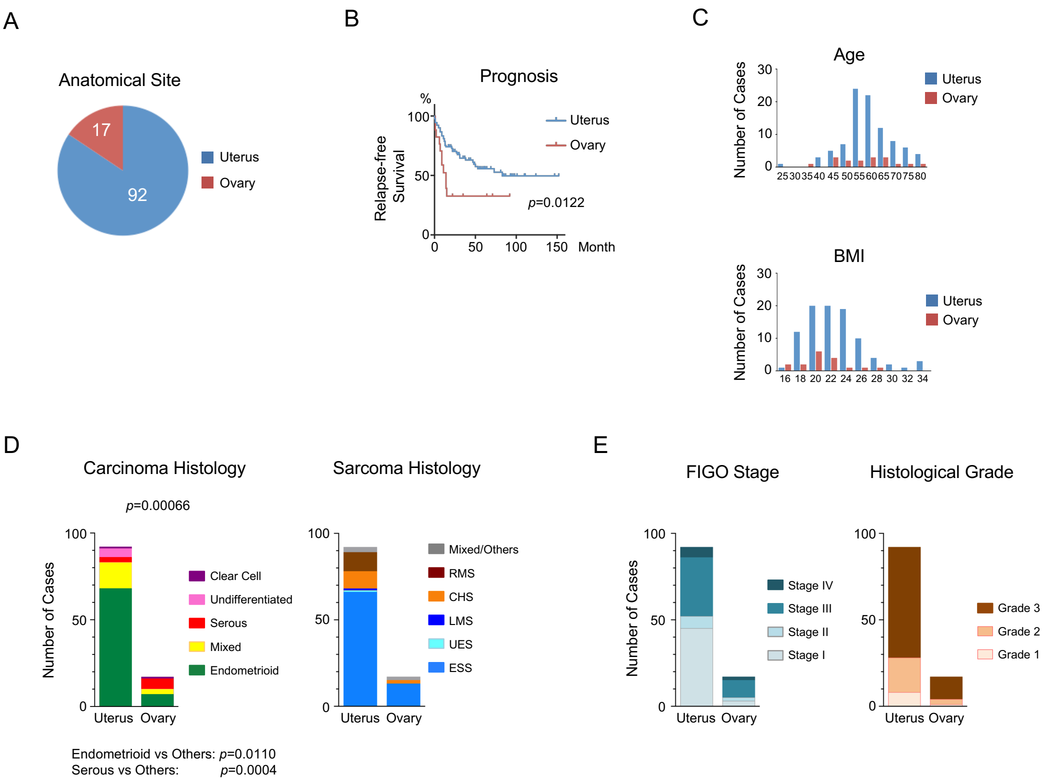

Supplementary Figure 1. Background information of carcinosarcoma (CS) samples in the current study.

A. Anatomical site of the primary tumor (uterus, navy blue; ovary, dark red). B. Prognosis of uterine and ovarian CS patients. Patients with ovarian CS had poorer prognosis than those with uterine CS (uterus, navy blue; ovary, dark red). C. Histograms for age (upper panel) and body mass index (BMI; lower panel) of patients with CS in the current cohort (uterus, navy blue; ovary, dark red). D. Histology of carcinoma (left panel) and sarcoma (right panel) components. Carcinoma histology was endometrioid (green), serous (red), clear cell (violet), undifferentiated (pink), and mixed (yellow). Histopathological diagnosis of homologous sarcomas was endometrial stromal sarcoma (ESS; sky blue), undifferentiated endometrial sarcoma (UES; light blue), or leiomyosarcoma (LMS; blue). Heterologous sarcomas were rhabdomyosarcoma (RMS; brown), chondrosarcoma (CHS; yellowish brown), mixed histology and other sarcomas (grey). E. Stage (left panel) and histological grade (right panel) of samples. Staging was performed according to the FIGO 2008 staging system. The color codes in the figure indicate stages and histological grades.

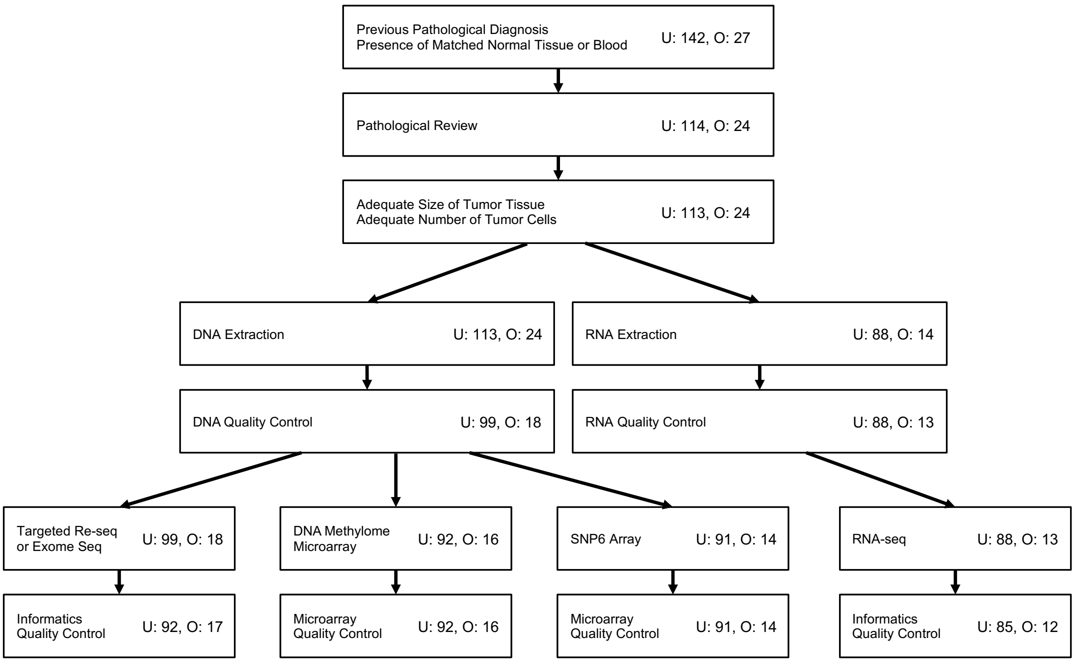

Supplementary Figure 2. REMARK diagram of the current study.

The sample numbers for primary bulk uterine and ovarian carcinosarcomas are shown at each step for pathological review, sample preparation, sequencing or microarray assays, and informatics or data quality control. Starting from 142 uterine and 27 ovarian CS primary tumors, 109 (92 uterine and 17 ovarian), 108 (92 uterine and 16 ovarian), 105 (91 uterine and 14 ovarian) and 97 (85 uterine and 12 ovarian) samples finally passed the stringent quality assessments during these steps for targeted re-sequencing or exome sequencing, DNA methylome, SNP6 arrays and RNA-seq analyses, respectively. Abbreviations: U, uterus; O, ovary.

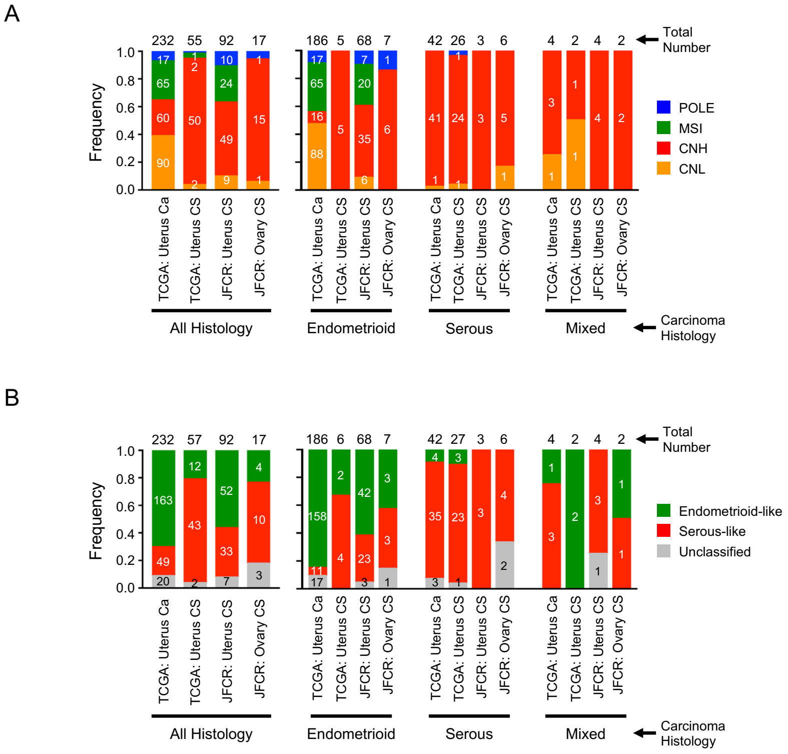

Supplementary Figure 3. Molecular subtyping of carcinosarcoma (CS) samples based on genomic aberration profiles for the 4 major driver mutations.

A. Genomic aberration subtypes of TCGA uterine endometrial endometrioid carcinomas, TCGA uterine CSs, and uterine and ovarian CSs in the current cohort (JFCR: Japanese Foundation for Cancer Research). Left: classification for all histology. Right: classification for endometrioid, serous, and mixed histology in the carcinoma component. Note that 2 TCGA uterine CSs were unable to be classified because they lacked the information necessary for genomic aberration subtyping. B. Driver mutation subtypes of TCGA uterine endometrial endometrioid carcinomas, TCGA uterine CSs, and uterine and ovarian CSs in the JFCR cohort. Two pairs of gynecological driver genes *PTEN* and *ARID1A* for “endometrioid-like”, and *TP53* and *PPP2R1A* for “serous-like” cancers were used for the molecular classification. Left: classification for all histology. Right: classification for endometrioid, serous, and mixed histology in the carcinoma element.

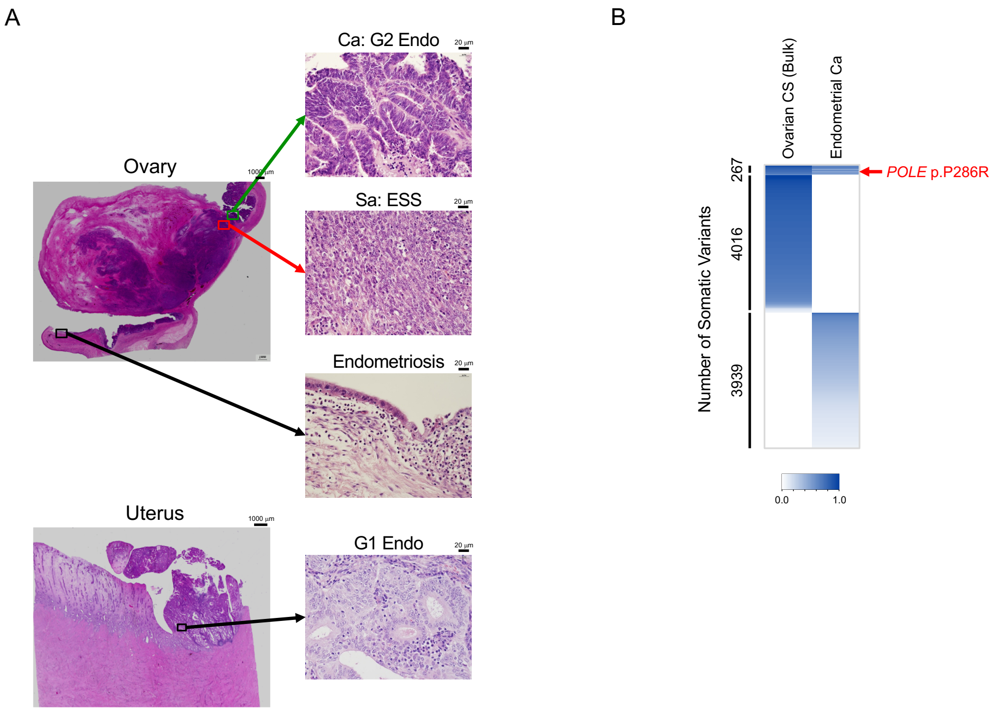

Supplementary Figure 4. Histological and exome analyses of synchronous primary cancers of the uterus and ovary in an ovarian POLE CS case (OV594).

A. Histopathology of synchronous ovarian and endometrial cancers. Left panels: The macroscopic images of the ovarian CS with associated endometriosis (upper left panel) and of the endometrial endometrioid carcinoma (lower left panel). Right panels from top to bottom: Microscopic images at high-power magnification (400×) of carcinoma (grade-2 endometrioid) and sarcoma (endometrial stromal sarcoma) components in CS, associated endometriosis, and synchronous grade-1 endometrial endometrioid carcinoma. Sections were stained with hematoxylin and eosin (HE). Scale bars are indicated with length in µm above the image. B. Heatmaps of mutant allele frequencies (MAFs) of somatic SNVs and indels in ovarian CS and endometrial endometrioid carcinoma. MAFs were adjusted by the tumor content and are shown as a heatmap after sorting by frequency. *POLE* p. P286R mutation is marked on the right. Note that 267 among a total of 8,222 somatic variants, including the *POLE* mutation, were genomically shared between the synchronous primaries. Numbers of ovarian and endometrial specific variants were 4,016 and 3,939, respectively. Abbreviations: Ca, carcinoma component; Sa, sarcoma component; G1, grade 1; G2, grade 2; Endo, endometrioid; and ESS, endometrial stromal sarcoma. See also Supplementary Table 1.

The ovarian CS tumor was associated with an endometriotic lesion in the ovary. The endometrial tumor (grade-1 endometrioid carcinoma), which was histologically distinct from the carcinoma component (grade-2 endometrioid) of the ovarian CS, synchronously existed in the uterine corpus. In accordance with previously proposed criteria [3], this case was diagnosed as synchronous endometrial and ovarian cancer (SEOC). Through exome sequencing analyses of the ovarian CS and the synchronous endometrial carcinoma, we show that both tumors shared a subset of SNVs/indels, including POLE p. P286R mutation. This observation is consistent with the findings from the previous NGS analyses, in which most pairs of primary tumors in SEOC patients shared relevant driver events, indicating the synchronous primaries were usually clonally derived [4, 5]. In view of this association with endometriosis, we concluded that this ovarian POLE CS of OV594 was likely to have originated from the endometria and likely shares ancestry with the synchronous endometrial carcinoma.

A

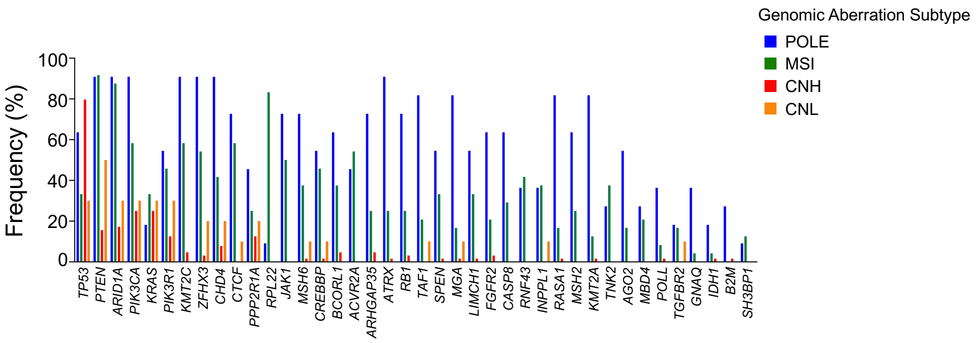

B

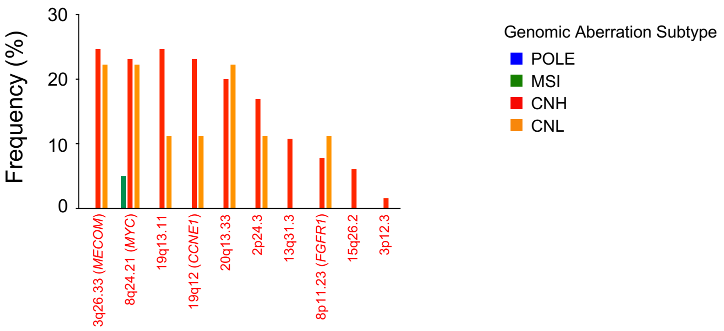

Supplementary Figure 5. Subtype specificity of driver genes.

A. Frequencies of somatic SNV and indel drivers for each subtype. Frequencies of driver genes per subtype are shown with different colored bars (POLE, blue; MSI, green; CNH, red; CNL, orange). The same set of driver genes as that in Figure 3A is shown. B. Frequencies of somatic CNV drivers for each subtype. Frequencies of driver genes per subtype are shown with different colored bars (POLE, blue; MSI, green; CNH, red; CNL, orange). The same set of driver genes as that in Figure 3B is shown.

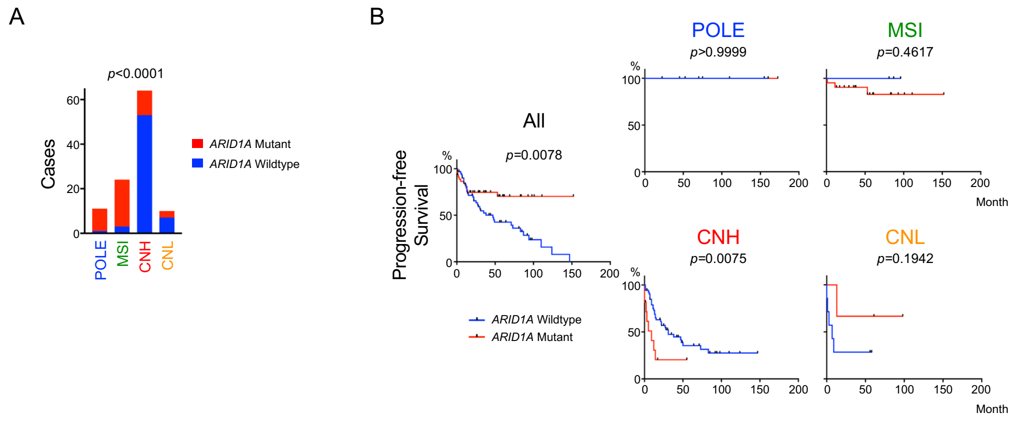

Supplementary Figure 6. Effect of *ARID1A* mutation on patient prognosis.

A. *ARID1A* mutation in genomic aberration subtypes. Status of *ARID1A* mutation is shown as a stacked bar plot. The p-value was computed using Fisher exact test with a 2 (presence or absence of *ARID1A* mutation) by 4 (each genomic aberration subtype) contingency table. B. Kaplan–Meier analyses for each genomic aberration subtype comparing *ARID1A* mutant and wildtype. Kaplan–Meier curves are shown with p-values computed by the log-rank test. Note that the correlation between *ARID1A* mutation positivity and favorable outcomes from "all" subtype patients was the reverse of that for those with poorer prognosis when the analysis was limited to CNH cases.

A

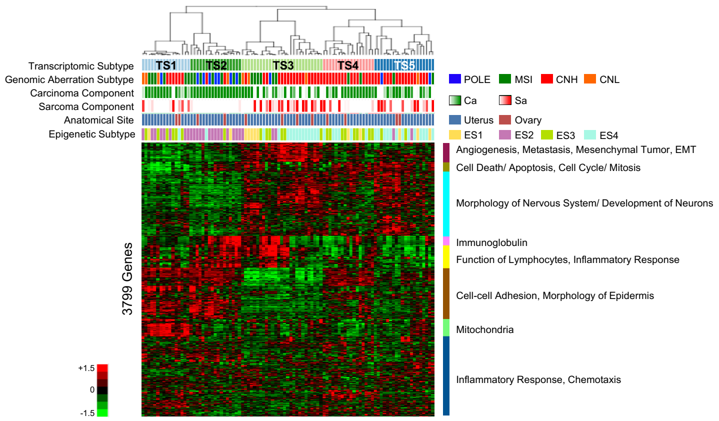

B

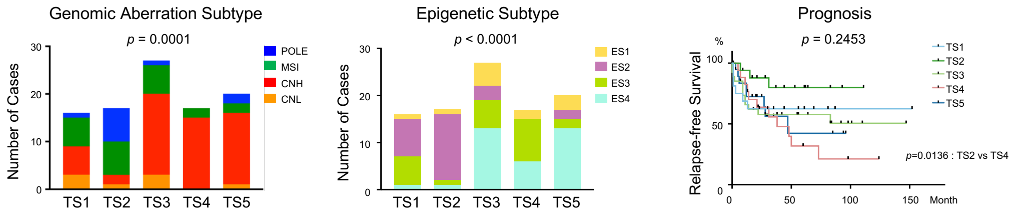

Supplementary Figure 7. Transcriptomic CS subtypes.

A. Gene expression heatmap for transcriptomic subtypes. Five transcriptomic subtypes (TSs: TS1, turquoise; TS2, green; TS3, bright green; TS4, coral red; and TS5, cobalt blue) identified with *K*-means consensus clustering of 3,799 genes with variable expression across the samples, are shown. Red and green indicate the high and low expression of genes, respectively. The results from the gene ontology analyses with Ingenuity Pathway Analysis are shown beside the heatmap. Color codes indicate genomic aberration subtypes (POLE, blue; MSI, green; CNH, red; and CNL, orange), the content of carcinoma and sarcoma components (green and red gradients, respectively), the anatomical site of the primary tumors (uterus, navy blue; ovary, dark red), and the epigenetic subtypes (ES1, yellow; ES2, purple; ES3, light green; ES4, sky blue). B. Left. The relationship between transcriptomic subtypes and genomic aberration subtypes (POLE, blue; MSI, green; CNH, red; and CNL, orange). Middle. The relationship between transcriptomic subtypes and epigenetic subtypes (ES1, yellow; ES2, purple; ES3, light green; and ES4, sky blue). Right. Kaplan–Meier curves for transcriptomic subtypes. Although there was no significant difference among the 5 subtypes, pairwise analyses identified a significant difference between TS2 and TS4 (p = 0.0136).

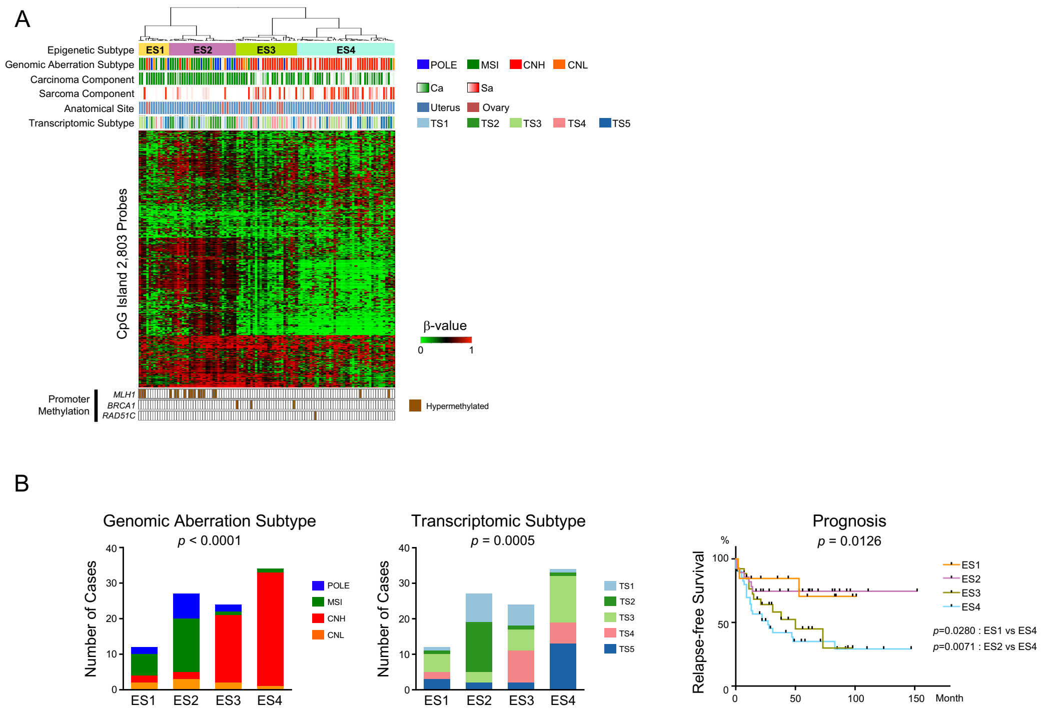

Supplementary Figure 8. Epigenetic CS subtypes.

A. DNA methylation heatmap for epigenetic subtypes. Four epigenetic subtypes (ESs: ES1, yellow; ES2, purple; ES3, light green; ES4, sky blue), identified with *K*-means consensus clustering of 2,803 probes with differentially methylated CpG islands across the samples, are shown. Green and red colors in the heatmap indicate β values for hypo- and hyper-methylated promoter CpG islands, respectively. Hypermethylation status for *MLH1* and *BRCA1* promoter CpG islands are shown at the bottom. A β value of 0.4 was used as a threshold for hypermethylation. Color codes indicate genomic aberration subtypes (POLE, blue; MSI, green; CNH, red; and CNL, orange), the content of carcinoma and sarcoma components (green and red gradients, respectively), the anatomical site of the primary tumors (uterus, navy blue; ovary, dark red) and the transcriptomic subtypes (TS1, turquoise; TS2, green; TS3, bright green; TS4, coral red; and TS5, cobalt blue). B. Left. The relationship between epigenetic subtypes and genomic aberration subtypes (POLE, blue; MSI, green; CNH, red; and CNL, orange). Middle. The relationship between epigenetic subtypes and transcriptomic subtypes (ES1, yellow; ES2, purple; ES3, light green; and ES4, sky blue). Right. Kaplan–Meier curves for transcriptomic subtypes. There were significant differences among the 4 epigenetic subtypes (p = 0.0126), between ES1 and ES4 (p = 0.0280), and between ES2 and ES4 (p = 0.0071).

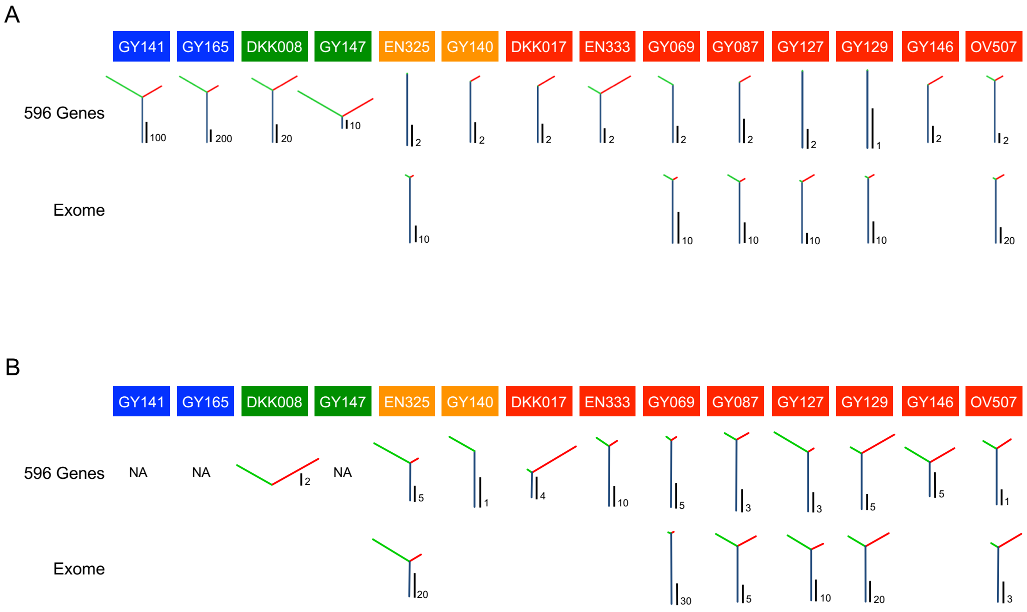

Supplementary Figure 9. Clonal evolution in the carcinoma and sarcoma elements inferred from sequenced data.

A. Clonal evolution inferred from SNVs/indels. Upper panels: SNVs/indels based on 596-gene panel. Note that these trees are the same as those in Figure 5. Lower panels: SNVs/indels based on exome. B. Clonal evolution inferred from CNVs. Upper panels: CNVs based on 596-gene panel. Lower panels: CNVs based on exome. CNVs were computed from the EXCAVATOR analysis of the sequenced data. Phylogenetic trees are used to represent genetic similarity of the carcinoma and sarcoma components in the primary tumor. A trunk (navy) and a branch (yellow green; carcinoma and cherry color; sarcoma) represent shared and private mutations, respectively. The length of each trunk and branch is proportional to the number of SNVs/indels and CNVs, and an index beside a tree indicates the number of base pairs and abnormal segments, respectively. A case ID is shown on each tree with the color indicating the genomic aberration subtypes (blue, POLE; green, MSI; red, CNH; and orange, CNL). Among 14 tumors with selective sampling of carcinoma and sarcoma components, 6 tumor data were acquired with exome sequencing. Therefore, for these 6 tumors, we also estimated the clonal evolution processes with SNVs/indels and with CNVs using exome data. The phylogenetic tree based on CNV information could not be computed for GY141, GY165, and GY147 cases because no aberrant segments were detected in the carcinoma or sarcoma elements. Abbreviations: NA, not available.

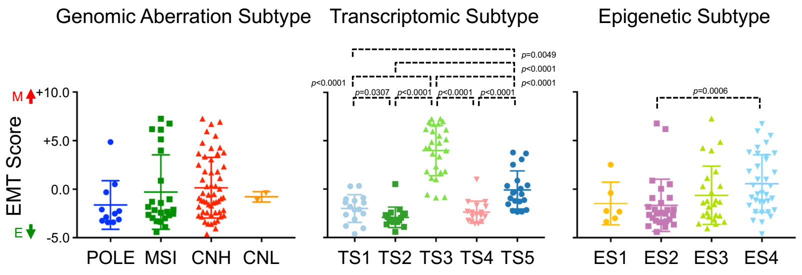

Supplementary Figure 10. EMT scores in molecular, transcriptomic, and epigenetic subtypes. EMT scores were plotted for the molecular (genomic aberration; left panel), transcriptomic (middle panel), and epigenetic (right panel) subtypes. Significant *p*-values are shown after statistical analyses with pairwise Mann–Whitney *U*-tests. Bars and error bars indicate mean and standard deviation.

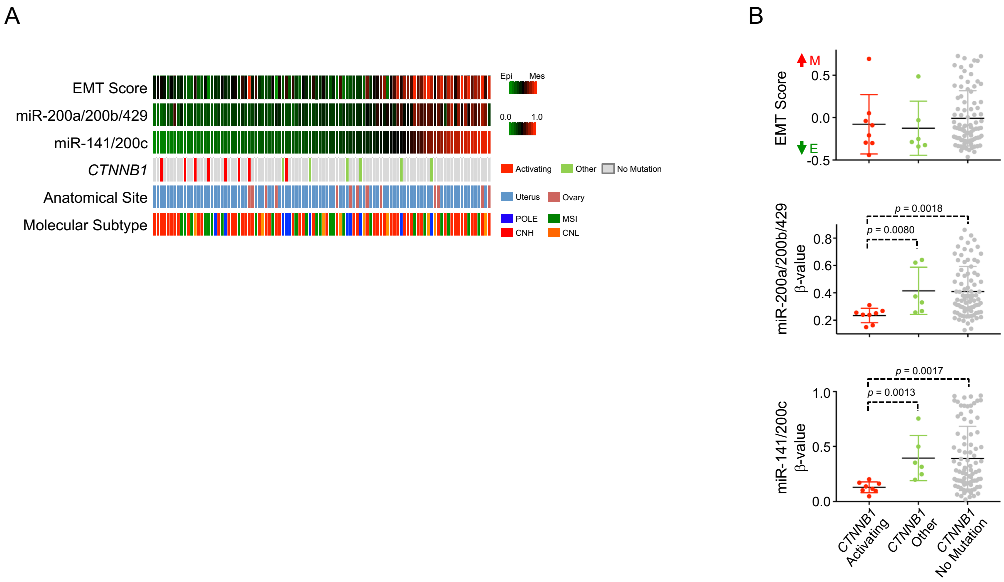

Supplementary Figure 11. Relationship among EMT score, methylation status of CpG sites for miR-141/200c and miR-200a/200b/429, and mutational status of *CTNNB1*.

A. Heatmap presentations of EMT scores and β values of CpG sites for miR-141/200c and miR-200a/200b/429 with mutational status of *CTNNB1*. Mutational status of *CTNNB1* (activating mutation, red; the other mutation, light green; and no mutation, grey), primary tumor anatomical site (uterus, navy blue; ovary, dark red), and genomic aberration subtype (POLE, blue; MSI, green; CNH, red; CNL, orange) data are presented below the heatmaps. B. Dot plot presentations of EMT score (upper panel) and β values of CpG sites for miR-141/200c (middle panel) and miR-200a/200b/429 (lower panel) per the mutational status of *CTNNB1*. Significant *p*-values are shown (Mann–Whitney *U*-tests).

Supplementary Tables

Supplementary Table 1: Synchronous Endometrial and Ovarian Cancer in the Current Cohort.

Case ID	Site of Primary Tumor	Size (mm)	Histology	FIGO Stage	Carcinoma Grade	Note
OV343	Left Ovary	110 × 23 × 10	Carcinosarcoma (Endometrioid+ESS)	IV	3	
	Right Ovary	19 × 15 × 6	Endometrioid Carcinoma	IC	3	Endometriosis-associated
	Uterine Corpus	18 × 10 × 2	Endometrioid Carcinoma	IA	3	
OV594	Right Ovary	106 × 90 × 52	Carcinosarcoma (Endometrioid+ESS)	IA	2	Endometriosis-associated
	Uterine Corpus	10 × 9 × 8	Endometrioid Carcinoma	IA	1	

Abbreviations: ESS; Endometrial Stromal Sarcoma

Supplementary Table 2: Sample Information with Selectively Sampling.

Case ID	Primary Site	Tissue Processing FFPE or FF	Sample ID	Separate Sampling Carcinoma or Sarcoma	Molecular Subtype	Exome Sequencing	Target Panel Sequencing
DKK008	Uterus	FFPE	DKK008T1	Carcinoma	MSI	-	+
			DKK008T2	Sarcoma	MSI	-	+
DKK017	Uterus	FFPE	DKK017T1	Carcinoma	CNH	-	+
			DKK017T2	Sarcoma	CNH	-	+
EN325	Uterus	FF	EN325T2	Carcinoma	CNL	+ (v5)	-
			EN325T3	Sarcoma	CNL	+ (v5)	-
EN333	Uterus	FFPE	EN333T1	Carcinoma	CNH	-	+
			EN333T2	Sarcoma	CNH	-	+
GY069	Uterus	FF	GY069T5	Carcinoma	CNH	+ (v5)	-
			GY069T6	Sarcoma	CNH	+ (v5)	-
GY087	Uterus	FF	GY087T2	Carcinoma	CNH	+ (v5)	-
			GY087T3	Sarcoma	CNH	+ (v5)	-
GY127	Uterus	FF	GY127T1	Carcinoma	CNH	+ (v5)	-
			GY127T2	Sarcoma	CNH	+ (v5)	-
GY129	Uterus	FF	GY129T2	Carcinoma	CNH	+ (v5)	-
			GY129T3	Sarcoma	CNH	+ (v5)	-
GY140	Uterus	FFPE	GY140T1	Carcinoma	CNL	-	+
			GY140T2	Sarcoma	CNL	-	+
GY141	Uterus	FFPE	GY141T1	Carcinoma	POLE	-	+
			GY141T2	Sarcoma	POLE	-	+
GY146	Uterus	FFPE	GY146T1	Carcinoma	CNH	-	+
			GY146T2	Sarcoma	CNH	-	+
GY147	Uterus	FFPE	GY147T1	Carcinoma	MSI	-	+
			GY147T2	Sarcoma	MSI	-	+
GY165	Uterus	FFPE	GY165T1	Carcinoma	POLE	-	+
			GY165T2	Sarcoma	POLE	-	+
OV507	Ovary	FFPE	OV507T1	Carcinoma	CNH	+ (v5)	-
			OV507T2	Sarcoma	CNH	+ (v5)	-

Abbreviations: FFPE: Formalin Fixed Paraffin Embedded; FF: Fresh Frozen

Supplementary Table 3: Sample Information with Multi-regional Sampling.

Case ID	Primary Site	Tissue Processing FFPE or FF	Molecular Subtype	Sample ID	Exome Sequencing	Target Panel Sequencing
EN482	Uterus	FFPE	MSI	EN482T1	+ (v5)	-
				EN482T2	+ (v5)	-
				EN482T3	+ (v5)	-
				EN482T4	+ (v5)	-
				EN482T5	+ (v5)	-
				EN482T6	+ (v5)	-
EN558	Uterus	FFPE	CNL	EN558T1	+ (v5)	-
				EN558T2	+ (v5)	-
				EN558T3	+ (v5)	-
				EN558T4	+ (v5)	-
				EN558T5	+ (v5)	-
				EN558T7	+ (v5)	-
				EN558T8	+ (v5)	-
EN676	Uterus	FFPE	POLE	EN676T1	+ (v5)	-
				EN676T2	+ (v5)	-
				EN676T4	+ (v5)	-
GY030	Uterus	FFPE	CNH	GY030T1	+ (v5)	-
				GY030T2	+ (v5)	-
				GY030T3	+ (v5)	-
				GY030T4	+ (v5)	-
				GY030T6	+ (v5)	-
				GY030T7	+ (v5)	-
				GY030T8	+ (v5)	-

Abbreviations: FFPE: Formalin Fixed Paraffin Embedded; FF: Fresh Frozen

Supplementary Table 4: Number of clonal or subclonal, driver or passenger, and trunk or branch mutations derived from carcinoma and sarcoma components in a tumor.

	Genomic Aberration Subtype	POLE		MSI	
	Sample ID	GY141	GY165	DKK008	GY147
Trunk	Clonal Driver	4	17	6	3
	Subclonal Driver	9	13	2	1
	Clonal Passenger	60	426	41	7
	Subclonal Passenger	147	380	10	5
Branch	Clonal Driver	0	0	0	2
	Subclonal Driver	10	16	1	9
	Clonal Passenger	32	10	15	31
	Subclonal Passenger	268	712	43	75

	Genomic Aberration Subtype	CNH								CNL	
	Sample ID	DKK017	EN333	GY069	GY087	GY127	GY129	GY146	OV507	EN325	GY140
Trunk	Clonal Driver	1	4	4	2	4	1	4	3	1	3
	Subclonal Driver	1	0	1	0	0	0	2	0	3	2
	Clonal Passenger	2	0	2	1	0	0	0	6	3	1
	Subclonal Passenger	2	3	0	2	4	1	1	5	2	0
Branch	Clonal Driver	0	0	0	0	0	0	0	0	0	0
	Subclonal Driver	0	0	0	0	0	0	0	0	0	0
	Clonal Passenger	1	0	0	0	0	0	0	0	0	0
	Subclonal Passenger	1	7	2	1	0	0	2	4	0	1

Trunk: SNV/indels shared by carcinoma and sarcoma elements.
Branch: SNV/indels specific for carcinoma or sarcoma element.
Clonal and subclonal mutations refer to mutations with ≥ 0.8 and < 0.8 cancer cell fractions.
Driver genes are 40 genes identified through IntOGen analysis (Figure 3) and passenger genes are the other genes.

Supplementary Table 5: Number of Clonal or Subclonal, Driver or Passenger, and Trunk, Shared Branch or Private Branch Mutations Derived from Multi-regional Samples in a Tumor.

A. EN676 (POLE)

	Trunk	Branch			
		Shared	Private		
	T1T2T4	T1T2	T1	T2	T4
Clonal Driver	3	0	0	0	0
Subclonal Driver	9	31	10	1	2
Clonal Passenger	163	212	254	3	86
Subclonal Passenger	897	6285	2463	922	804

B. EN482 (MSI)

	Trunk	Branch									
		Shared				Private					
	T1T3T2T6T4T5	T1T3	T2T6T4T5	T2T6T4	T2T6	T1	T3	T2	T6	T4	T5
Clonal Driver	1	5	2	0	0	0	0	0	0	0	2
Subclonal Driver	2	1	1	1	0	2	0	1	3	0	2
Clonal Passenger	22	199	115	139	2	11	89	0	3	5	72
Subclonal Passenger	7	85	45	128	56	305	177	105	191	187	246

C. GY030 (CNH)

	Trunk	Branch									
		Shared			Private						
	T1T2T3T4T6T7T8	T1T2T3T4T6T7	T2T3	T4T6	T1	T2	T3	T4	T6	T7	T8
Clonal Driver	1	0	0	0	0	0	0	0	0	0	0
Subclonal Driver	1	0	0	0	0	0	0	0	0	0	0
Clonal Passenger	8	0	0	0	0	0	0	0	0	0	2
Subclonal Passenger	40	10	2	3	18	6	21	18	12	27	28

D. EN558 (CNL)

	Trunk	Branch									
		Shared			Private						
	T1T8T2T4T3T5T7	T1T8T2T4T3	T1T8	T2T4	T1	T8	T2	T4	T3	T5	T7
Clonal Driver	2	0	0	0	0	0	0	0	0	0	0
Subclonal Driver	1	0	0	0	0	0	0	0	0	0	0
Clonal Passenger	50	2	0	0	0	0	0	0	0	0	0
Subclonal Passenger	20	10	4	3	21	11	15	19	10	26	15

Each of T1, T2, T3,--- indicates the sequenced region (Supplementary Figure 7).

Trunk: SNV/indels shared by carcinoma and sarcoma elements. Branch: SNV/indels specific for carcinoma or sarcoma element.

Clonal and subclonal mutations refer to mutations with ≥ 0.8 and < 0.8 cancer cell fractions.

Driver genes are 40 genes identified through IntOGen analysis (Figure 3) and passenger genes are the other genes.

Supplementary References

1. Liu Y*, et al.* Clinical significance of CTNNB1 mutation and Wnt pathway activation in endometrioid endometrial carcinoma. *J Natl Cancer Inst* **106**, (2014).
2. Cancer Genome Atlas Research N*, et al.* Integrated genomic characterization of endometrial carcinoma. *Nature* **497**, 67-73 (2013).
3. Scully RE, Young RH, Pathology AFIo, Clement PB, Research UAf, Pathology Ei. *Tumors of the Ovary, Maldeveloped Gonads, Fallopian Tube, and Broad Ligament*. Armed Forces Institute of Pathology under the auspices of Universities Associated (1998).
4. Anglesio MS*, et al.* Synchronous Endometrial and Ovarian Carcinomas: Evidence of Clonality. *J Natl Cancer Inst* **108**, djv428 (2016).
5. Schultheis AM*, et al.* Massively Parallel Sequencing-Based Clonality Analysis of Synchronous Endometrioid Endometrial and Ovarian Carcinomas. *J Natl Cancer Inst* **108**, djv427 (2016).
