# Supplementary material for: Clinically relevant molecular subtypes and genomic alteration-independent differentiation in gynecologic carcinosarcoma
Source: Nat Commun. 2019 Oct 31;10:4965. doi: 10.1038/s41467-019-12985-x (PMC6823358; doi:10.1038/s41467-019-12985-x)
Supplement: Supplementary file 3 — Description of Additional Supplementary Files [file 41467_2019_12985_MOESM3_ESM.pdf]

Description of Additional Supplementary Files

File Name: Supplementary Data 1
Description: Sample Information for Histology and Genomic Aberration Subtype.

File Name: Supplementary Data 2
Description: Samples and Analytical Methods.

File Name: Supplementary Data 3
Description: Source of Matched Normal Samples.

File Name: Supplementary Data 4
Description: Genes in the Target Panel.

File Name: Supplementary Data 5
Description: The Mutant Allele Frequency of SNVs/indels in 596 Genes in the Panel for Selectively Sampled Carcinoma and Sarcoma Elements from 14 Cases.

File Name: Supplementary Data 6
Description: The Copy Number of CNVs in 596 Genes in the Panel for Selectively Sampled Carcinoma and Sarcoma Elements from 14 Cases.
