# Supplementary material for: Clinically relevant molecular subtypes and genomic alteration-independent differentiation in gynecologic carcinosarcoma
Source: Nat Commun. 2019 Oct 31;10:4965. doi: 10.1038/s41467-019-12985-x (PMC6823358; doi:10.1038/s41467-019-12985-x)
Supplement: Supplementary file 10 — Reporting Summary [file 41467_2019_12985_MOESM10_ESM.pdf]

nature research

Corresponding author(s): Seiichi Mori

Last updated by author(s): Sep 19, 2019

Reporting Summary

Nature Research wishes to improve the reproducibility of the work that we publish. This form provides structure for consistency and transparency in reporting. For further information on Nature Research policies, see Authors & Referees and the Editorial Policy Checklist.

Statistics

For all statistical analyses, confirm that the following items are present in the figure legend, table legend, main text, or Methods section.

n/a	Confirmed	
☐	☒	The exact sample size (*n*) for each experimental group/condition, given as a discrete number and unit of measurement
☐	☒	A statement on whether measurements were taken from distinct samples or whether the same sample was measured repeatedly
☐	☒	The statistical test(s) used AND whether they are one- or two-sided<br>*Only common tests should be described solely by name; describe more complex techniques in the Methods section.*
☐	☒	A description of all covariates tested
☐	☒	A description of any assumptions or corrections, such as tests of normality and adjustment for multiple comparisons
☐	☒	A full description of the statistical parameters including central tendency (e.g. means) or other basic estimates (e.g. regression coefficient) AND variation (e.g. standard deviation) or associated estimates of uncertainty (e.g. confidence intervals)
☐	☒	For null hypothesis testing, the test statistic (e.g. *F*, *t*, *r*) with confidence intervals, effect sizes, degrees of freedom and *P* value noted<br>*Give P values as exact values whenever suitable.*
☒	☐	For Bayesian analysis, information on the choice of priors and Markov chain Monte Carlo settings
☒	☐	For hierarchical and complex designs, identification of the appropriate level for tests and full reporting of outcomes
☐	☒	Estimates of effect sizes (e.g. Cohen's *d*, Pearson's *r*), indicating how they were calculated

Our web collection on statistics for biologists contains articles on many of the points above.

Software and code

Policy information about availability of computer code

Data collection	Sequence data: CASAVA (v1.8.2); Epigenome data: Genome Studio (v2011.1)
Data analysis	Open source code/packages used for data analysis: R/Bioconductor packages, BWA (v0.6.1), GATK UnifiedGenotyper (v3.4.0), VarScan (v2.3.7), MuTect (v1.1.4), Karkinos (v3.0.22), SomaticIndelDetector (v1.5-30), EXCAVATOR (v2.2) , GISTIC (v2.0.22), IntOGen (v3.0.5), PHYLIP (v3.695), Inkscape (v0.92), Bowtie2 (v2.2.3) and RSEM (v1.3.0).<br>Commercial software used for data analysis: GraphPad Prism (v8) and Microsoft Excel for Mac (v16).

For manuscripts utilizing custom algorithms or software that are central to the research but not yet described in published literature, software must be made available to editors/reviewers. We strongly encourage code deposition in a community repository (e.g. GitHub). See the Nature Research guidelines for submitting code & software for further information.

Data

Policy information about availability of data

All manuscripts must include a data availability statement. This statement should provide the following information, where applicable:

- Accession codes, unique identifiers, or web links for publicly available datasets
- A list of figures that have associated raw data
- A description of any restrictions on data availability

Exome/target panel sequencing data have been deposited in the National Bioscience Database Center (NBDC; https://biosciencedbc.jp/en/) under the accession number JGAS00000000172 [https://ddbj.nig.ac.jp/jga/viewer/view/study/JGAS00000000172]. RNA sequencing data have been deposited in the NCBI Gene Expression Omnibus (GEO; https://www.ncbi.nlm.nih.gov/geo/) under the accession number GSE128630 [https://www.ncbi.nlm.nih.gov/geo/query/acc.cgi?acc=GSE128630]. The TCGA uterine CS dataset was accessed from the Genomic Data Commons [https://portal.gdc.cancer.gov/projects/TCGA-UCS]. DNA methylation data have been deposited in the NCBI Gene Expression Omnibus (GEO; https://www.ncbi.nlm.nih.gov/geo/) under the accession number GSE136790 [https://www.ncbi.nlm.nih.gov/geo/query/acc.cgi?acc= GSE136790].

Field-specific reporting

Please select the one below that is the best fit for your research. If you are not sure, read the appropriate sections before making your selection.

☒ Life sciences ☐ Behavioural & social sciences ☐ Ecological, evolutionary & environmental sciences

For a reference copy of the document with all sections, see nature.com/documents/nr-reporting-summary-flat.pdf

Life sciences study design

All studies must disclose on these points even when the disclosure is negative.

Sample size	Sample size was determined based on availability. To our knowledge, this is the largest gynecologic carcinosarcoma cohort with molecular data.
Data exclusions	The study cohort initially consisted of 169 (142 uterine and 27 ovarian) samples. Of these, 138 samples (114 uterine and 24 ovarian CSs) underwent further processing after being consistently diagnosed as CS according to the World Health Organization (WHO) 2014 classification by at least two independent pathologists with expertise in gynecological pathology. A total of 109 CS samples (92 uterine and 17 ovarian) finally passed the stringent quality assessments during sample preparation, sequencing, and informatics analyses for targeted re-sequencing or exome sequencing.
Replication	Not applicable; these are patient data.
Randomization	Observational study; no randomization was performed.
Blinding	No blinding was considered necessary.

Reporting for specific materials, systems and methods

We require information from authors about some types of materials, experimental systems and methods used in many studies. Here, indicate whether each material, system or method listed is relevant to your study. If you are not sure if a list item applies to your research, read the appropriate section before selecting a response.

Materials & experimental systems

n/a	Involved in the study	
☒	☐	Antibodies
☒	☐	Eukaryotic cell lines
☒	☐	Palaeontology
☒	☐	Animals and other organisms
☐	☒	Human research participants
☒	☐	Clinical data

Methods

n/a	Involved in the study	
☒	☐	ChIP-seq
☒	☐	Flow cytometry
☒	☐	MRI-based neuroimaging

Human research participants

Policy information about studies involving human research participants

Population characteristics	Clinicopathological features, such as anatomical site, carcinoma and sarcoma histology, stage, grade, and other patient variables, are shown in Supplemental Figure 1 and are described in the Supplemental Materials and Methods.
Recruitment	Carcinosarcoma patients underwent surgery at the Cancer Institute Hospital, Kyoto University Hospital, or Saitama Medical University International Medical Center between 1998 and 2015.
Ethics oversight	Ethical approval was obtained by the internal review boards of the JFCR, Kyoto University Hospital, and Saitama Medical University. Recruited patients were provided with written informed consent.

Note that full information on the approval of the study protocol must also be provided in the manuscript.
